# The regulation of FGF23 under physiological and pathophysiological conditions

**DOI:** 10.1007/s00424-022-02668-w

**Published:** 2022-01-27

**Authors:** Steffen Rausch, Michael Föller

**Affiliations:** Department of Physiology, University of Hohenheim, Garbenstraße 30, 70599 Stuttgart, Germany

**Keywords:** Klotho, Vitamin D, PTH, Phosphate, CKD

## Abstract

Fibroblast growth factor 23 (FGF23) is an important bone hormone that regulates phosphate homeostasis in the kidney along with active vitamin D (1,25(OH)_2_D_3_) and parathyroid hormone (PTH). Endocrine effects of FGF23 depend, at least in part, on αKlotho functioning as a co-receptor whereas further paracrine effects in other tissues are αKlotho-independent. Regulation of FGF23 production is complex under both, physiological and pathophysiological conditions. Physiological regulators of FGF23 include, but are not limited to, 1,25(OH)_2_D_3_, PTH, dietary phosphorus intake, and further intracellular and extracellular factors, kinases, cytokines, and hormones. Moreover, several acute and chronic diseases including chronic kidney disease (CKD) or further cardiovascular disorders are characterized by early rises in the plasma FGF23 level pointing to further mechanisms effective in the regulation of FGF23 under pathophysiological conditions. Therefore, FGF23 also serves as a prognostic marker in several diseases. Our review aims to comprehensively summarize the regulation of FGF23 in health and disease.

## Introduction

Fibroblast growth factor 23 (FGF23) was discovered as an endocrine factor produced in bone that may be considered as the missing link of the kidney-parathyroid gland-bone axis [9]. It helps maintain phosphate homeostasis not only by regulating parathyroid hormone (PTH) and 1,25(OH)_2_D_3_ (calcitriol), active vitamin D, secretion, but also by directly targeting renal phosphate transport [9]. Phosphate is essential for a bunch of cellular processes including nucleic acid production, energy metabolism, or signal transduction (phosphorylation/dephosphorylation of signaling molecules) [14]. Moreover, it is part of hydroxyapatite that makes up the essential inorganic compound of bone [14].

## FGF family

FGF23 is a relatively new protein in evolution [82]. The mammalian family comprises two types of FGFs: intracellular FGFs and extracellular FGFs [82]. FGFs 11–14 function in the cell as signaling molecules and play a role in neuronal excitability [82]. Extracellular FGFs can be subdivided into endocrine and canonical (also named paracrine) members depending on heparin or heparan sulfate as a cofactor [82]. Endocrine FGF15/19, 21, and 23 exhibit low affinity for heparin cofactors and therefore require Klotho proteins as co-receptors [82].

## FGF23

FGF23 (251 amino acids) displays the highest expression in bone (osteoblasts and osteocytes) but can also be detected in other organs including the liver, brain, heart, thyroid, intestine, and skeletal muscle [70, 87, 109]. As a prerequisite for its endocrine properties, it is devoid of the heparan-sulfate binding motif which would result in high extracellular matrix binding, allowing its secretion into blood [82]. FGF23 secretion is strongly influenced by posttranslational modification, i.e., O-glycosylation and phosphorylation [14]. The polypeptide N-acetylgalactosaminyltransferase 3 (GALNT3) O-glycosylates FGF23, resulting in its secretion and preventing its phosphorylation by family with sequence similarity 20 member C (FAM20C) which would lead to FGF23 breakdown [14]. Subtilisin-like proprotein convertases (SPC) cleave FGF23 at a certain motif leading to inactive C-terminal (25–179) and N-terminal (180–251) FGF23 residues [9]. Commercial ELISAs detecting C-terminal FGF23 (cFGF23) or uncleaved intact FGF23 (iFGF23) are commonly used for plasma samples. Possibly, cFGF23 is not only inactive, but may suppress FGF23 signaling [48]. FGF23 effects can be exerted in an αKlotho-independent or αKlotho-dependent fashion [48]. FGF23 receptors include fibroblast growth factor receptor (FGFR)1c, FGFR3c, and FGFR4 [82]. αKlotho binds to FGF23 thereby enhancing its receptor affinity [82].

## αKlotho

The relevance of αKlotho was discovered in 1997: Mice with markedly reduced αKlotho expression exhibit accelerated aging with multiple aging-associated diseases and die early [66]. In its transmembrane form, αKlotho is a co-receptor for FGF23 while soluble αKlotho has FGF23-independent paracrine and endocrine effects [9]. Soluble αKlotho is generated by cleavage of its extracellular domain or alternative splicing [29]. It regulates membrane proteins including ion channels and controls intracellular pathways such as insulin-like growth factor I or Wnt signaling [65].

## Effects of FGF23

**Fig. 1:**
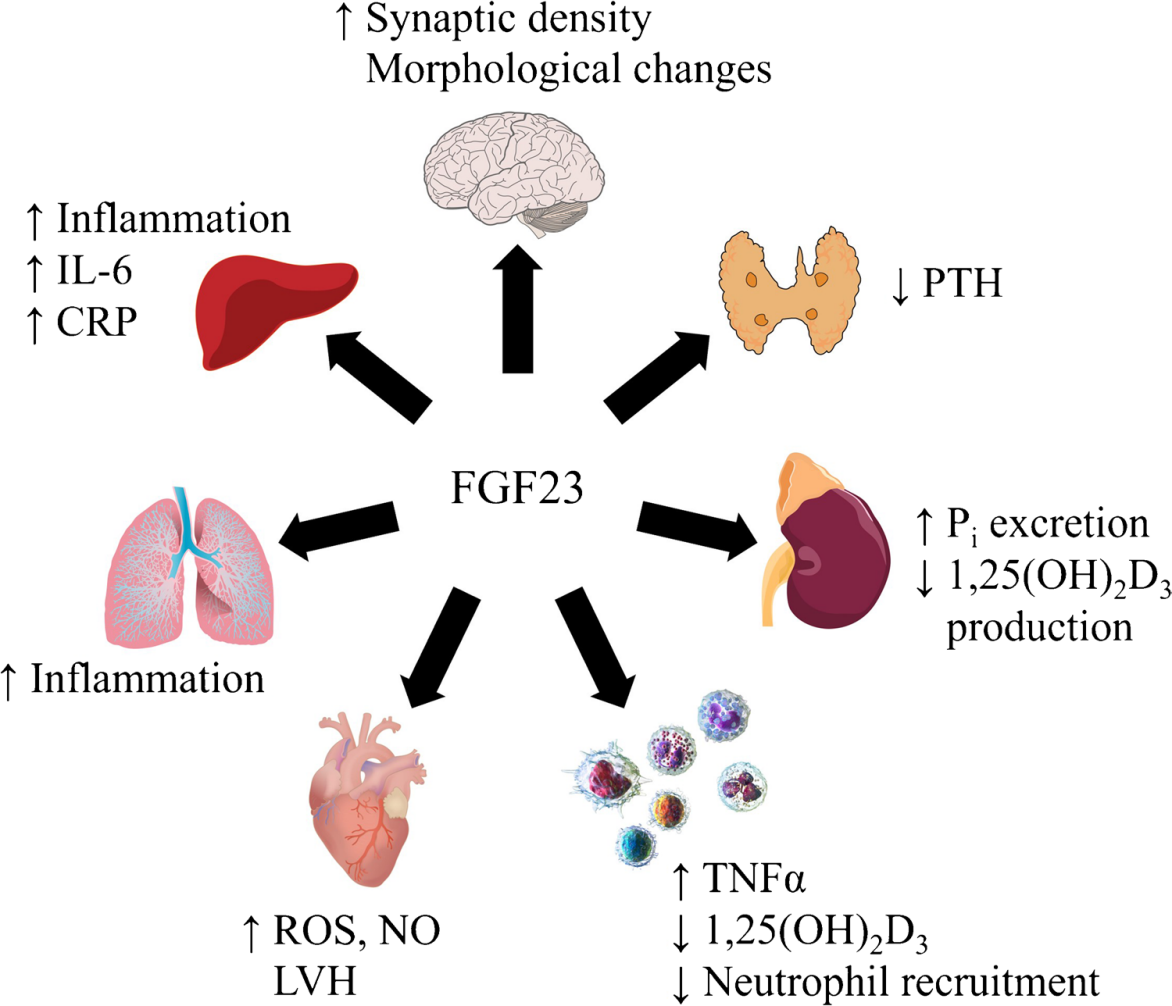
Effect of FGF23 in different organs and cells. C-reactive protein (CRP), fibroblast growth factor 23 (FGF23), inorganic Phosphate (Pi), interleukin-6 (IL-6), left ventricular hypertrophy (LVH), parathyroid hormone (PTH), reactive oxygen species (ROS), tumor necrosis factor α (TNFα). Sources: Heart: Injurymap, CC BY 4.0, Leukocytes: Blausen.com staff (2014). “Medical gallery of Blausen Medical 2014.” *WikiJournal of Medicine* 1 (2). DOI:10.15347/wjm/2014.010. ISSN 2002–4436., CC BY 3.0

The effects of FGF23 in different organs, tissues, and cells are displayed in Fig. 1.

### Kidney

**Fig. 2:**
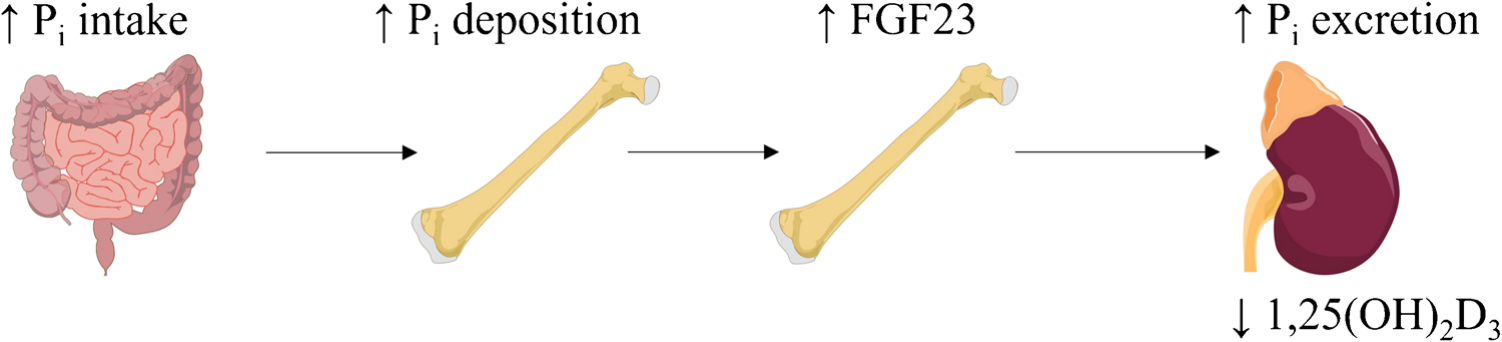
FGF23 is upregulated upon alimentary phosphate intake and regulates renal phosphate and vitamin D handling

FGF23 is a major regulator of phosphate homeostasis that is dependent on the interplay of different organs: Alimentary phosphate is absorbed in the intestine; most extracellular phosphate is deposited in bone; and the kidney is responsible for urinary excretion of phosphate that is filtered in the glomeruli [57] (Fig. 2). Moreover, PTH and 1,25(OH)_2_D_3_ are further regulators of phosphate homeostasis and FGF23 [9]. FGF23 induces renal phosphate excretion by decreasing surface expression of NaPiIIa and NaPiIIc, the major Na^+^-dependent phosphate transporters of the proximal tubule [57]. FGF23 downregulates renal *cytochrome P450 (Cyp)27b1* expression, the key enzyme for 1,25(OH)_2_D_3_ production, and enhances *Cyp24a1* production catalyzing the inactivation of 1,25(OH)_2_D_3_ [57]. These effects of FGF23 are αKlotho-mediated [29].

### Parathyroid glands

**Fig. 3:**
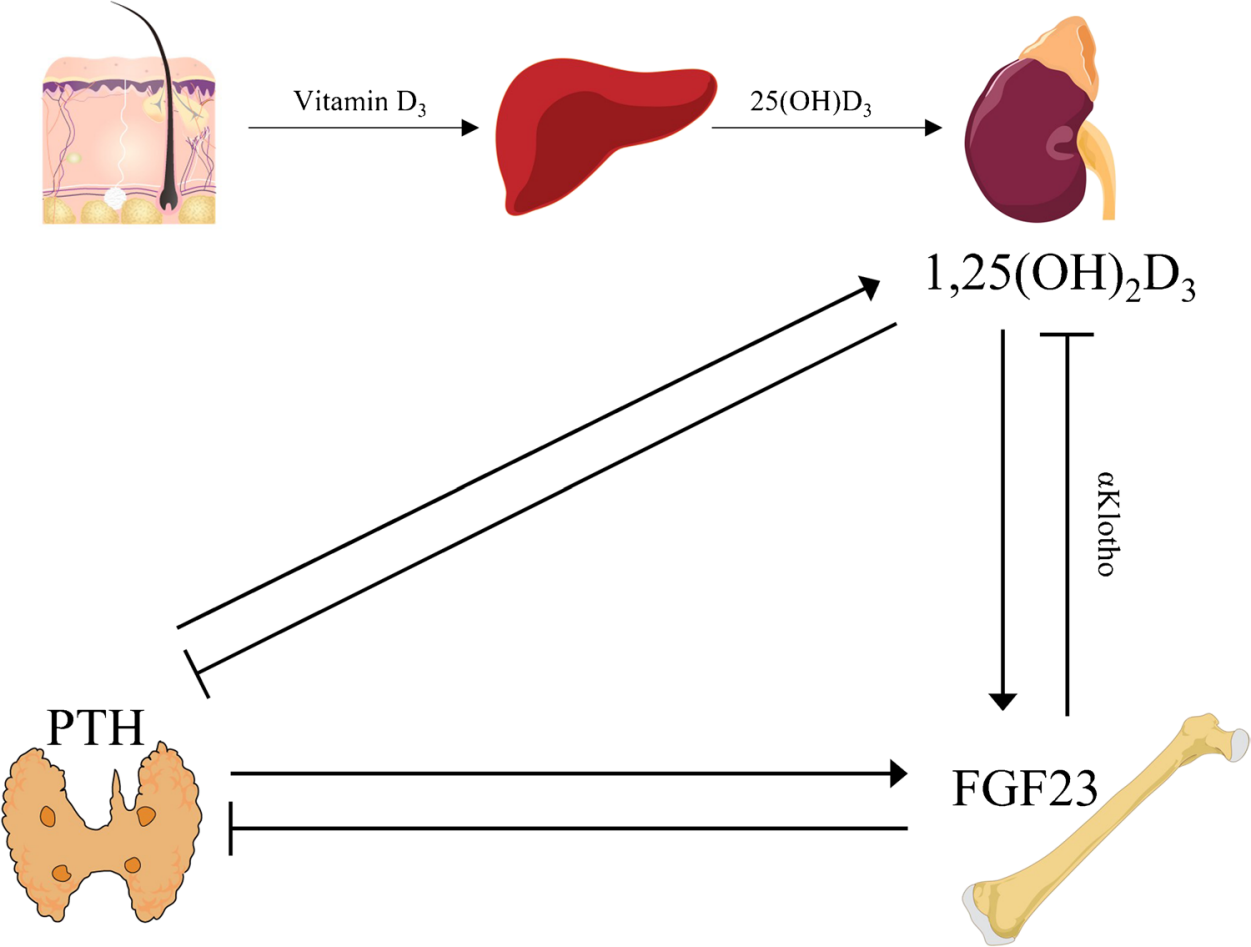
The interdependence of FGF23, PTH, and 1,25(OH)_2_D_3_. Source: Skin: DBCLS 統合TV, CC BY 4.0

FGF23 inhibits *Pth* expression and lowers PTH plasma levels through mitogen-activated protein kinase (MAPK) signaling and, in an αKlotho-independent manner, through calcineurin/nuclear factor of activated T-cells (NFAT) signaling [14, 75]. The interdependence of FGF23, PTH, and 1,25(OH)_2_D_3_ is summarized in Fig. 3.

### Bone

FGF23 controls bone mineralization [78].

### Brain

FGF23 increases synaptic density and changes morphology of hippocampal cells [53].

### Heart

FGF23 induces left ventricular hypertrophy (LVH) through FGFR4 [49]. In isolated cardiac myocytes, FGF23 favors pro-fibrotic signaling [68]. FGF23 stimulates NO synthesis and reactive oxygen species (ROS) generation in human coronary endothelial cells [89].

### Immune system

Lipopolysaccharide (LPS) and interferon γ (IFNγ) enhance *Fgf23* expression while FGF23 stimulates tumor necrosis factor α (TNFα) production in pro-inflammatory macrophages [50]. FGF23 suppresses 1,25(OH)_2_D_3_ production in monocytes [3] and interferes with neutrophil recruitment [91].

### Liver

FGF23 upregulates interleukin (IL)-6 and C-reactive protein (CRP) expression in the liver, thereby promoting inflammation in chronic kidney disease [96].

### Lung

In bronchial epithelial cells, FGF23 also stimulates inflammation [63].

### Muscle

Physical exercise enhances FGF23 production, and FGF23 increases mitochondrial function and helps cope with ROS production [70].

## Regulation of FGF23

In the following, we in an alphabetical order summarize intracellular and extracellular factors regulating gene expression, production, and secretion of FGF23 (Table 1).

**Table 1:** Regulators of FGF23

Factor	Influence on FGF23
1,25(OH)_2_D_3_	↑ [9]
Acidosis	↑ [64]
Actin cytoskeleton	↑ [36]
Advanced glycation endproducts	↑ [7]
Aldosterone	↑ [84, 113]
AMPK	↓ [47]
Cadmium	↑ [62]
Calcineurin inhibitors	↓ [5]
Calciprotein	↑ [1]
Calcium	↑ [19]
cFGF23	Inhibits signaling [48]
DMP1	↓ [25, 73]
Endothelin-1	↓ [39]
ENPP1	↓ [54]
ERR-γ	↑ [87]
Erythropoietin	↑ [44]
FGFR1 signaling	↑ [107]
Glucocorticoids	↓ [40]
HIF1α	↑ [104, 116]
High-fat diet	↑ [46]
IL-1β	↑ [59, 81, 110]
IL-6β	↑ [24]
Insulin	↓ [4]
Insulin-like growth factor	↓ [4]
Iron	↓ [52]
Lactic acid	↑ [2]
Leptin	↑ [102]
Lipocalin 2	↑ [17]
Lithium	↑ [37, 114]
LPS	↑ [81]
Lysophosphatidic acid	↑ [95]
Myostatin	↑ [32]
NF-κB	↑ [2, 7, 33, 114, 115]
Nurr1	↑ [75]
p38MAPK	↑ [33]
PHEX	↓ [8, 111]
Phosphate	↑ [9, 55]
PKC	↑ [6]
Plasminogen activation	↓ [30]
PPARα	↓ [34]
Propranolol	↓ [35]
PTH	↑ [75, 81]
SOCE	↑ [34, 41, 47, 114, 115]
Sympathetic activity	↑ [35, 61]
TGF-β2	↑ [41]
TNFα	↑ [46, 81]
Vitamin A	↓ [88]

### Actin cytoskeleton

Reorganization of the actin cytoskeleton controlled by Rac1/PAK1 signaling is a prerequisite for *Fgf23* expression in vitro [36].

### Autonomic nerve system

The circadian rhythm governs sympathetic activity which enhances FGF23 production [61]. During the dark phase, *Fgf23* expression goes up in bone [61]. This regulation is dependent on cryptochrome 1 [61]. In mice with a GSK3 mutation rendering it insensitive to PKB/Akt/SGK signaling, enhanced sympathetic activity is associated with elevated FGF23 serum levels [35]. The latter are lowered by β-adrenergic receptor blocker propranolol [35].

### Calcineurin inhibitors

Ca^2+^-dependent phosphatase calcineurin inhibitors tacrolimus and ciclosporin A are widely used as immunosuppressants and inhibit *Fgf23* gene expression in vitro [5].

### Calcium

Hypocalcemia is associated with low FGF23 levels as a study of Gcm2^−/−^ mice characterized by hypocalcemia, hyperphosphatemia, and low calcitriol and PTH levels and Cyp27b1^−/−^ mice with hypocalcemia, hypophosphatemia, and low 1,25(OH)_2_D_3_ but high PTH levels has revealed [19]. Conversely, a high-calcium diet increases FGF23 serum concentration in the transgenic mice without affecting 1,25(OH)_2_D_3_ or PTH, pointing to an independent role of extracellular Ca^2+^ in regulating FGF23 [19]. Store-operated Ca^2+^ entry (SOCE) through Ca^2+^ release-activated calcium channel protein 1 (Orai1) in conjunction with Ca^2+^-sensing protein STIM1 is part of the cellular machinery enhancing *Fgf23* transcription in vitro [115]. Calciprotein particles composed of calcium, phosphate, and fetuin-A also stimulate *Fgf23* expression [1].

### C-Term FGF23

C-terminal FGF23 inhibits FGF23 signaling by impeding formation of the αKlotho FGFR1c complex in vivo and in vitro [48].

### Endothelins

Endothelin-1 (ET-1) reduces FGF23 production through endothelin B receptor (ETB) in vitro and in vivo [39].

### Energy metabolism

Insulin and insulin-like growth factor 1 suppress FGF23 production in vitro and in vivo [4]. This effect is mediated by induction of PI3K/PKB/Akt activity inhibiting transcription factor FOXO1 [4]. Consequently, insulin-deficient mice are characterized by elevated FGF23 serum concentrations that is decreased by insulin administration [4]. In a human study, a negative correlation of plasma insulin and FGF23 was found [4]. Cellular energy sensor 5′-adenosine monophosphate (AMP)-activated kinase (AMPK) is activated in energy deficiency and inhibits FGF23 production in vivo and in vitro through suppression of Orai1-mediated SOCE [47]. Fibrates, agonists of lipid metabolism-associated transcription factor PPARα, downregulate FGF23 in vitro, an effect at least partly mediated by AMPK-dependent regulation of SOCE [34]. Adipokine leptin induces *Fgf23* expression in vivo [102]. Acidosis is associated with enhanced FGF23 production [64]. Moreover, lactic acid concentrations encountered in severe lactic acidosis upregulate *Fgf23* expression in vitro, an effect at least in part dependent on nuclear factor kappa-light-chain-enhancer of activated B cells (NF-κB) signaling [2]. Advanced glycation endproducts induce *Fgf23* gene expression in an NF-κB-dependent manner [7].

### ENPP1

In autosomal recessive hypophosphatemic rickets type 2 (ARHR2), ectonucleotide pyrophosphatase/phosphodiesterase family member 1 (ENPP1) fails to keep FGF23 levels low due to inactivating mutations in the ENPP1 gene resulting in hypophosphatemia [54].

### ERR-γ

Orphan nuclear estrogen-related receptor-γ (ERR-γ) increases hepatic FGF23 synthesis in acute kidney injury (AKI) [87].

### DMP1

Dentin matrix acidic phosphoprotein 1 (DMP1) is a protein produced by osteoblasts and osteocytes and regulates the mineralization of extracellular matrix [25]. In vivo, DMP1 deficiency is associated with enhanced *Fgf23* expression with hypophosphatemia [73], and in vitro DMP1 downregulates FGF23 through NFAT signaling [25].

### G-3-P

Glycerol-3-phosphate (G-3-P) released in AKI is positively correlated with FGF23 levels in humans and enhances *Fgf23* transcription in bone [95]. This effect is dependent on G-3-P acyltransferases converting G-3-P to lysophosphatidic acid that activates LPA receptor 1 in vitro [95].

### Inflammation

As a mediator of inflammation-dependent upregulation of FGF23, pro-inflammatory IL-1β elevates FGF23 serum levels through bone resorption [110] and through enhanced gene expression in vitro [59]. Also, pro-inflammatory IL-6 directly stimulates *Fgf23* expression through STAT3 signaling [24]. TNFα enhances FGF23 production in chronic inflammation [26] and in mice upon high-fat diet feeding [46]. An enhancer element 16 kb upstream of the start site of *Fgf23* gene transcription accounts for LPS-, IL-1β-, TNF-α-, and PTH-induced *Fgf23* expression [81]. NF-κB is a prominent transcription factor complex involved in pro-inflammatory responses [115]. In vitro, NF-κB induces *Orai1* expression, facilitating SOCE which enables *Fgf23* transcription [115]. Lipocalin 2 (LCN2) is an iron chelator and part of innate immune responses [17]. In CKD, it stimulates FGF23 production, at least in part through cAMP signaling [17].

### Iron, EPO, and HIF1α

In mice, iron deficiency results in upregulated *Fgf23* expression and iFGF23 as well as cFGF23 serum levels [52], an effect involving hypoxia inducible factor 1 α (HIF1α) [104] which is a transcriptional regulator of FGF23 [116]. HIF1α target erythropoietin (EPO) also stimulates FGF23 production [44].

### Kinases

P38 mitogen-activated protein kinase (p38MAPK) is activated upon exposure of cells to stress and stimulates *Fgf23* expression in vitro, an effect at least in part depending on NF-κB [33].

### Metal ions

Cadmium impacts on post-translational modification of FGF23, stimulating its secretion in vitro and in vivo [62]. This effect requires p38MAPK-dependent activation of aryl hydrocarbon receptor leading to enhanced GALNT3 production [62]. Lithium stimulates FGF23 production in vitro and in vivo through NF-κB-dependent Orai1 and SOCE regulation [37, 114].

### Nurr1

Nuclear receptor-associated protein1 (Nurr1) mediates PTH-dependent upregulation of *Fgf23* expression in vitro and in vivo [75].

### Paracrine/autocrine FGFR1 signaling

Regulation of FGFR1 signaling through autocrine and paracrine FGFs influences *Fgf23* transcription, an effect involving PLCγ, MAPK, and PI3K/Akt signaling [107].

### PHEX

Loss of PHEX activity elevates plasma FGF23 levels, as typical of X-linked hypophosphatemia (XLH) [8]. This effect is dependent on PHEX enhancing FGF23 degradation through SPC or PHEX-DMP1-integrin complexes [111].

### Phosphate

Phosphate induces *Fgf23* transcription through ROS in vitro [55].

### PKC

In vitro, protein kinase C (PKC) activation through phorbol ester enhances whereas PKC inhibition downregulates *Fgf23* gene expression [6].

### Plasminogen activation

Overexpression of plasminogen activator inhibitor-1 (PAI-1) elevates FGF23 levels in mice whereas tissue-type and urokinase-type plasminogen activators cleave FGF23 in vitro [30].

### Steroid hormones

Anti-inflammatory glucocorticoids suppress *Fgf23* expression in vitro and FGF23 serum levels in mice, at least in the short term [40]. Mineralocorticoid aldosterone upregulates *Fgf23* transcription in vitro and in vivo [84, 113]. In Klotho deficiency, enhanced 1,25(OH)_2_D_3_ leads to extracellular volume depletion which further worsens outcome [43].

### TGF-β

Transforming growth factor-β2 (TGF-β2) upregulates *Fgf23* transcription and secretion through SOCE in vitro [41]. Myokine myostatin also stimulates *Fgf23* expression and secretion in vitro [32].

### Vitamin A

Retinoic acid receptor (RAR) signaling induced by vitamin A compounds inhibits *Fgf23* expression and protein secretion in vitro [88].

## Pathophysiological roles of FGF23

The pathophysiological role of FGF23 is not limited to diseases with hypophosphatemia or hyperphosphatemia. Also, further acute and chronic disorders not associated with altered phosphate metabolism are characterized by changes in the plasma FGF23 concentration.

### Acute kidney injury

Acute kidney injury leads to increased FGF23 levels [87, 95].

### Airway inflammation

In chronic obstructive pulmonary disease, FGF23 is elevated [63].

### Autosomal dominant polycystic kidney disease

Patients with autosomal dominant polycystic kidney disease are mainly characterized by high cFGF23 and, in part also, high iFGF23 levels [85]. In rodent models of this disease, iFGF23 levels are elevated [97].

### Cancer

Rare forms of colon adenocarcinoma are characterized by FGF23 secretion with hypophosphatemia [67] whereas in other forms, plasma FGF23 is increased [60]. In urothelial cancer, FGF23 is also elevated [71]. Further malignancies found to exhibit, at least in part, higher FGF23 levels are ovarian cancer [101], prostate cancer [42], and multiple myeloma [99]. For further review, see [31].

### Cardiovascular disease

**Fig. 4:**
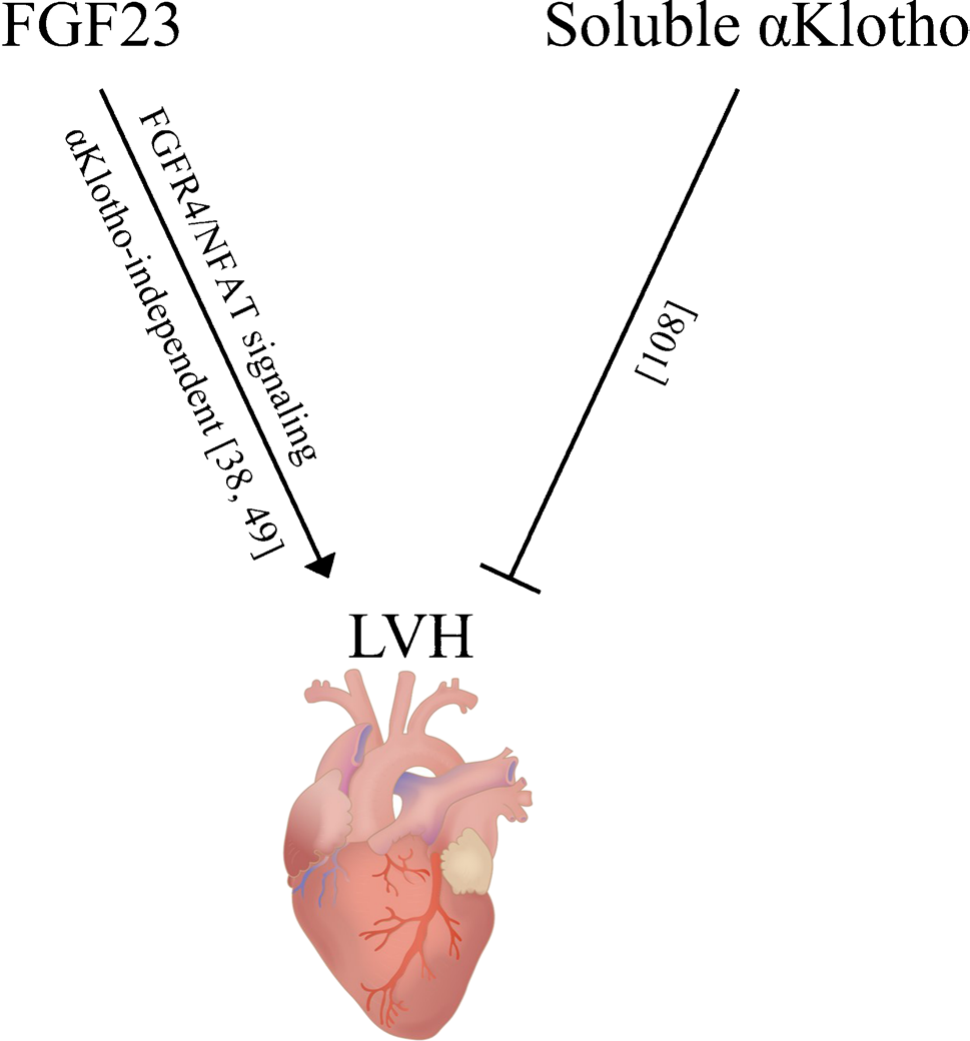
Effect of FGF23 and αKlotho in the heart. Heart: Injurymap, CC BY 4.0

FGF23 induces LVH without αKlotho in mice [38]. However, Klotho deficiency also induces LVH without involvement of FGF23 [108]. Interestingly, cardiac *Fgf23* overexpression in healthy mice does not cause LVH, supporting a role of αKlotho or phosphate status in the progression of LVH [69]. Due to these results, the exact role of FGF23 in heart disease remains somewhat controversial (Fig. 4) [98]. In human cohorts, FGF23 is positively associated with left ventricular heart mass in CKD patients [38]. In patients with coronary artery disease, higher FGF23 levels are associated with increased risk of death [83]. In CKD patients and in the elderly, increased levels of iFGF23 are positively correlated with aortic calcification [20, 76, 79]. Higher FGF23 levels are associated with atrial fibrillation in CKD [74]. High FGF23 is also a risk factor for myocardial infarction, hemorrhagic stroke [22], and heart failure [21].

### CKD

CKD is often characterized by hyperphosphatemia due to failure of the kidney to excrete phosphate [56]. As hyperphosphatemia is a major trigger of enhanced FGF23 secretion, high FGF23 plasma levels are typical of CKD [103]. However, since FGF23 goes up early in CKD prior to the onset of hyperphosphatemia or hyperparathyroidism [58], other factors including inflammation are also effective [26]. FGF23 is a reliable prognostic marker in CKD correlating with outcome [45]. Upon kidney transplantation, cFGF23 is correlated with graft loss [16]. In CKD patients, higher abundance of oxidized PTH is observed [112]. In contrast to non-oxidized PTH, oxidized PTH is not correlated with plasma FGF23, and in vitro, oxidized PTH is less capable of inducing *Fgf23* gene expression [112]. Moreover, in CKD, the positive association of plasma Klotho with GFR is absent in patients with high FGF23 levels [93].

### Diabetes and obesity

FGF23 levels are positively associated with increased insulin resistance and obesity [51].

### Hyperphosphatemic disorders

Hyperphosphatemic familial tumoral calcinosis type 1–3 (HFTC) is characterized by hyperphosphatemia, normal or high calcitriol levels, and phosphate retention [11]. It is due to loss of function mutation in the gene encoding GALNT3 (type I), FGF23 (type II), and αKlotho (type III) ultimately causing FGF23 deficiency or resistance to FGF23 [11].

PTH-dependent hyperphosphatemic disorders include pseudohypoparathyroidism, where PTH resistance causes a decrease of 1,25(OH)_2_D_3_ and an increase in serum FGF23 concentration [117].

### Hypophosphatemic disorders

Autosomal dominant hypophosphatemic rickets (ADHR) is due to mutations rendering FGF23 resistant to cleavage [94]. In tumor-induced osteomalacia, tumor cells — often but not exclusively benign mesenchymal tumors — secrete FGF23 [15], resulting in hypophosphatemia as a hallmark. XLH is also caused by an abnormally high FGF23 plasma concentration that is due to loss-of-function mutations of the PHEX gene [100]. Inactivating mutations in the DMP1/ENPP1/FAM20C genes are responsible for ARHR1/2/3 with elevated FGF23 levels [57]. Fibrous dysplasia/McCune-Albright syndrome is caused by an activating mutation of GNAS resulting in high cAMP and FGF23 levels [10]. Activating mutations of PTH/PTHrP receptor gene account for Jansen’s metaphyseal chondrodysplasia characterized by high FGF23 plasma concentration [12]. Activating mutations of FGFR1 gene are the reason for osteoglophonic dysplasia characterized by high FGF23 levels and hypophosphatemia [105]. Increased αKlotho levels also result in hypophosphatemic rickets and increased iFGF23 plasma concentration [13].

### Inflammatory diseases

In inflammatory diseases, a correlation of inflammatory activity and plasma FGF23 is observed (e.g., rheumatoid arthritis [92], inflammatory bowel disease [28], sepsis in CKD patients [23]). In CKD, a higher FGF23 plasma concentration is correlated with higher inflammatory activity [77]. Since inflammation also contributes to CKD, it may contribute to the rise in plasma FGF23 typical of this disease [18].

### Iron deficiency

In the absence of CKD, iron deficiency is associated with an elevation of cFGF23 [106]. In general, treatment of iron deficiency with intravenous iron lowers cFGF23 on a transcriptional level while ferric carboxymaltose increases iFGF23 due to an inhibitory effect on its degradation [106]. In patients on dialysis, ferric carboxymaltose, however, decreases iFGF23 while elevating cFGF23 [90]. Upon renal transplantation, iron deficiency also drives an increase in cFGF23 and contributes to the poorer outcome of iron deficiency in CKD [27].

### Liver disease

In patients with end stage liver disease, FGF23 is increased owing to hepatic FGF23 production [86].

## Anti-FGF23 therapy

Burosumab is an antibody against FGF23 that is approved and therapeutically used in the treatment of X-linked hypophosphatemia [72]. Further FGF23-associated diseases for which anti-FGF23 therapy is tested include tumor-induced osteomalacia [80].

## Conclusions

FGF23 is part of a complex network with a very high degree of interdependence of the constituting regulating factors. Better understanding of the regulation of FGF23 is of high interest in view of the many pathologies impacting on the plasma FGF23 concentration. The endocrine effects of FGF23 are nowadays well established. However, the multiple paracrine effects in different tissues are less well studied. Moreover, the regulation of FGF23 under both, physiological and pathophysiological conditions is ill-defined including transcriptional and post-transcriptional mechanisms. In particular, it is not yet clear in many cases whether the increase in plasma FGF23 concentration observed in many diseases only indicates disease or whether FGF23 actively contributes to disease progression as observed in the heart. Also the role of anti-FGF23 therapy needs to be investigated. Definitely, further research is warranted.
